# Decontamination of patient equipment: nurses’ self-reported decontamination practice in hospitals of southeast Ethiopia

**DOI:** 10.1186/s13104-019-4427-5

**Published:** 2019-07-12

**Authors:** Biniyam Sahiledengle

**Affiliations:** Department of Public Health, Madda Walabu University Goba Referral Hospital, Bale-Goba, Ethiopia

**Keywords:** Decontamination, Disinfection, Sterilization, Reusable medical equipment, Nurses, Ethiopia

## Abstract

**Objective:**

Failure to adequately decontaminate patient equipment will increase the risk of transmission of infection between patients and may contribute to the development of hospital-acquired infections. In effect, full obedience towards the acceptable decontamination process by healthcare workers is required. The aim of this study was to assess decontamination practice and associated factors among nurses in hospitals of the southeast, Ethiopia.

**Results:**

A total of 273 nurses participated in the study with a response rate of 98.9%. Of these respondents, the acceptable decontamination practice was found to be 49.1% [95% CI 43.2–54.9%]. Nurses who have reported good infection prevention practice were 7.313 times more likely to had acceptable decontamination practice than there counterpart [AOR = 7.313; 95% CI: 4.030, 13.272, *p* value = 0.000]. Nurses who were working in the department having instructive posters or guideline target on instrument processing were 2.675 times more likely to had acceptable decontamination practice [AOR = 2.675; 95% CI: 1.376, 5.200, p-value = 0.004]. This low decontamination practice among nurses is a concern and might make hospitalized patients prone to different pathogenic microorganisms, which in turn can increase the risk of healthcare-associated infections. Therefore, enhancing the current nurses’ decontamination practice through considering those identified factors is crucial.

**Electronic supplementary material:**

The online version of this article (10.1186/s13104-019-4427-5) contains supplementary material, which is available to authorized users.

## Introduction

The use of physical or chemical means to remove, inactivate, pathogens on a surface or item to the point where they are no longer capable of transmitting infectious particles and the surface or item is rendered safe for handling, use, or disposal is termed as decontamination [1, 2]. Decontamination is the combination of processes, including cleaning, disinfection and/or sterilization, used to make a re-usable item safe for further use on patients [2–4].

Cleaning is the act of removing visible organic residue (e.g. blood and tissue) and inorganic salts from patient care equipment and preparing it for safe handling and/or further decontamination. Cleaning also removes sufficient numbers of microorganisms to reduce risks for those who touch or handle the object [3, 5, 6]. In addition, it has been highlighted as a serious responsibility and a critical factor in the battle against HAIs [7]. Disinfection is thermal or chemical destruction of most pathogenic and other types of microorganisms, but not all bacterial spores [3, 6]. Whereas sterilization destroys all microorganisms (bacteria, viruses, fungi, and parasites) including bacterial endospores from inanimate objects by high-pressure steam (autoclave), dry heat (oven), chemical sterilants or radiation [6, 8, 9]. In this regard, strict compliance with the recommended decontamination process at all level is required [9–12]. Since failure to properly disinfect or sterilize equipment carries not the only risk associated with a breach of host barriers but also the risk for person-to-person transmission (e.g., hepatitis B virus) and transmission of environmental pathogens (e.g., *Pseudomonas aeruginosa*) [3].

In Ethiopia, rigorous decontamination of reusable medical equipment is an important part of infection prevention and patient safety strategy [8]. However, the effects of the effort have not been felt across the country studies have shown that healthcare workers have sub-optimal infection prevention practice in Ethiopia [10, 13–15]. In addition, contamination of medical equipment with potential pathogens like *S. aureus, Klebsiella* spp.*, Citrobacter* spp.*, Salmonella* spp.*, Proteus* spp.*, Enterobacter* spp.*, P. aeruginosa, and E. coli* is reported [16]. Moreover, concerns have been raised as a result of the high prevalence of HAIs [17–19]. Given the reality that poor decontamination of patient equipment can result in a range of infections and contributing factor for HAIs [3, 20].

So far different studies were conducted in the area of infection prevention in Ethiopia [13–15, 21]. However, none of the previously conducted studies assessed the decontamination practice of healthcare workers particularly among nursing staffs; where decontamination most commonly performed by nurses in hospital settings in Ethiopia. The only available study in the country on instrument processing practice does not include hospitals [10]. And it identified factors that influenced decontamination practice such as; high-risk perception, knowledgeable on instrument processing, attitude towards infection prevention, and presence of SOP or guideline in workplace targeted on instrument decontamination [10]. Even though these factors are from a single study conducted in the health centers, my study has included and attested the majority of the variables in the hospital setting.

As far as I know, in southeast Ethiopia, there is no available information with regard to the issues of medical equipment decontamination practice among nurses. As a result, assessment of the current reusable medical equipment decontamination practice is timely. Therefore, the aim of this study was to assess decontamination practice and associated factors among nurses in hospitals of the southeast, Ethiopia.

## Main text

### Study design, area and period

A hospital-based cross-sectional study was conducted from April 15 to May 15/2018 among nurse’s found in at Bale zone hospitals, Southeast Ethiopia. In this study, all five hospitals (namely Goba referral hospital, Ginnir general hospital, Robe general hospital, Dellomena general hospital and Madda Walabu primary hospital) found in Bale zone were included.

### Sampling and participation

The source population was all nurses found in five Bale zone public hospitals. Selected nurses who work at least 6 months in the direct care of patients in Bale zone public hospitals in each department and case team was the study population. To determine the sample size for the study, Epi Info version 7.1.1.14 software using a single population proportion formula with the assumptions of 95% confidence level, 5% precision and prevalence of (p = 50%) acceptable decontamination practice were considered (since there was no study conducted in the study area). The calculated sample size was (n = 384). Since the source population (N = 641) was less than 10,000, it needed a finite population correction and the final sample size (nf) of the study was nf = 276, including a 15% non-response rate. First, the calculated sample size was proportionally allocated to each hospital, proportional to the number of nurses employed. Afterward, a computer-generated random number was used to select nurses from each hospital by using the list of all nurses in each hospital as a sampling frame.

### Data collection tool and procedure

A self-administered structured and pre-tested questionnaire was used to collect the data. The questionnaires were prepared after reviewing related literature [3, 6, 9, 10]. It was first prepared in English and then translated to Amharic and Afan Oromo (local languages) and back-translated to English to ensure consistency. Five 3rd year health officer students were recruited for data collection and two B.Sc. nurses for supervisor were employed. Data on socio-demographic characteristics, healthcare facility and behavioral related factors, awareness on instrument decontamination, and reusable medical equipment decontamination practice were collected.

### Data quality control

In order to enhance the quality of data, a pre-test was done prior to data collection on 5% of the actual sample size. In addition, the data collection tool was tested for internal consistency (reliability) using Cronbach’s alpha test. The resulting Cronbach’s Alpha value of 0.741 was obtained. During the data collection period, the questionnaires were checked on a daily basis for completeness.

### Data processing and analysis

Data were checked for completeness and consistency before data entry. The cleaned data were coded and entered into Epi-Data 3.1 software and analysis was done using SPSS version 20.0 statistical software. Descriptive statistics such as frequencies, percentages, and summary statistics (mean and standard deviation) were computed. Binary logistic regression analysis was employed to determine the crude association between the outcome and independent variables. Before putting variables into the multivariable logistic regression multicollinearity between the independent variables was tested. Lastly, multivariable logistic regression analysis was applied to describe the functional independent predictors of acceptable decontamination practice. For all statistical significant tests p-value < 0.05 was used as a cut-off point. Hosmer–Lemeshow goodness of fit test was also used for model checking.

### Operational definition

#### Decontamination practice

It has consisted of ten decontamination questions such as wear appropriate personal protective equipment while performing decontamination process, immediately soak contaminated equipment in 0.5% chlorine solution for 10 min, regularly monitor sterilization process, and correct labeling of the decontaminating solution was considered. Nurses who had considered “acceptable practice” if they respond seven and above (≥ 75%) items unless considered as “unacceptable practice”.

### Results

#### Socio-demographic characteristics of study participants

In this study, 273 nurses participated in the study with the response rate of 98.9%. Among the study participants, 131 (48%) participants were females. Majority of the respondents 239 (87.5%) did not receive any training targeted on instrument decontamination (Table 1).

**Table 1:** The socio-demographic, work and behavioral related characteristics of study participants in Bale zone hospital in 2018 (n = 272)

Variables	Category	Frequency (n = 272)	Percent (%)
Age (years)	< 30	192	70.3
	≥ 30	81	29.7
Sex	Male	142	52.0
	Female	131	48.0
Educational status	1st degree	153	56.0
	Diploma	120	44.0
Marital status	Married	156	57.1
	Single	117	42.9
Service year in the current hospital (years)	< 5	120	44.0
	≥ 5	153	46.0
Ever had training on instrument decontamination	Yes	34	12.5
	No	239	87.5
Received supportive supervise	Yes	209	76.6
	No	64	23.4
Current working department	Gynecology and obstetric ward, and operating room	88	32.2
	Surgical and medical ward	85	31.1
	Pediatrics	38	13.9
	Outpatient department (OPD) and other^a^	62	22.7

^a^Maternal and child health, Family planning, Emergency

#### Work and behavioral related characteristics of the study participants

Out of the total participants, 85 (31.1%) participants were from the surgical and medical department. Majority of the study participants, 209 (76.6%) had received supportive supervision. Only thirty-four (12.5%) of participants ever had training on instrument decontamination (Table 1).

#### Awareness on instrument decontamination

Based on this study, 169 (61.9%) participants knew instrument processing is one of the basic components of standard precaution practice and one hundred (36.6%) knew cleaning is the first step in instrument processing. Majority of the participants, 240 (87.9%) had awareness on how to prepare 0.5% decontaminant chlorine solution (Additional file 1: Table S1).

#### Reusable medical equipment decontamination practice

In this study, almost half 49.1% [95% CI 43.2–54.9%] of the study participants only had acceptable decontamination practice (Table 2).

**Table 2:** Self-reported reusable medical equipment decontamination practice among nurses in Bale zone hospitals in 2018 (n = 272)

Variables	Response	Frequency	Percent (%)
Did you use all necessary PPE when you perform instrument processing (n = 273)	Yes	196	71.8
	No	77	28.2
Did you use Google/eye shield while you perform instrument processing for the last time (n = 196)	Yes	42	21.4
	No	154	78.6
Did you use heavy duty glove when you handle, clean and process contaminated medical equipment for the last time (n = 196)	Yes	171	87.2
	No	25	12.8
Did you use a mask when you clean and/or decontaminate reusable medical equipment for the last time (n = 196)	Yes	57	29.1
	No	139	70.9
When do you soak contaminated items after any medical or surgical procedure	Immediately	49	17.9
	Within 30 min	193	70.7
	Within 1 h	31	11.4
	I don’t soak at all	0	0
Did you always soaked contaminated equipment in 0.5% chlorine solution before you perform cleaning (n = 273)	Yes	211	77.3
	No	62	22.7
How long did you soak contaminated equipment in 0.5% chlorine solutions the last time (n = 211)	5 min and less	0	0
	10 min	163	77.2
	For 1 h	44	20.9
	For 24 h	4	1.9
	I don’t know	0	0
Do you regularly monitor the steam and/or dry sterilized (autoclaves) using chemical indicators (n = 273)	Yes	54	19.8
	No	219	80.2
Do you regularly monitor the steam and/or dry sterilized (autoclaves) using biological indicators at least every week (n = 273)	Yes	0	0
	No	273	100
Do you always label the type, time and date of decontaminating solution (n = 273)	Yes	179	65.6
	No	94	34.4
Overall decontamination practice	Acceptable	134	49.1
	Unacceptable	139	50.9

#### Factors associated with decontamination practice

The Hosmer and Lemeshow test for the overall goodness of fit was used to check the correctness of the final formulated model and the value became 0.994 that is insignificant, which means the final fitted model was correct. In multivariable analysis, nurses who have reported good infection prevention practice were 7.313 times more likely to have acceptable decontamination practice than nurses who had poor infection prevention practice [AOR = 7.313; 95% CI: 4.030, 13.272, p-value = 0.000]. Participants who were working in the department having instructive posters or guideline target on instrument processing were almost 3 more likely to have acceptable decontamination practice than their counterparts [AOR = 2.675; 95% CI: 1.376, 5.200, p-value = 0.004] (Table 3).

**Table 3:** Factors associated with reusable medical equipment decontamination practice

Variables	Decontamination practice		COR (95% CI)	p-value	AOR	p-value
	Acceptable (n = 134)	Not acceptable (n = 139)				
Sex						
Male	66	76	0.805 (0.500–1.295)	0.370		
Female	68	63				
Educational status						
1st degree	73	80	0.883 (0.547–1.424)	0.609		
Diploma	61	59				
Awareness on decontamination						
Yes	91	78	1.655 (1.010–2.712)*	0.045	1.310 (0.722–2.380)	0.375
No	43	61				
IP training						
Yes	18	16	1.193 (0.581–2.450)	0.631		
No	116	123				
Receive supportive supervision						
Yes	114	95	2.640 (1.457–4.784)*	0.001	1.107 (0.504–2.431)	0.800
No	20	44	1		1	
Age						
20–29	84	108	0.482 (0.284–0.820)*	0.007	0.790 (0.381–1.640)	0.527
≥ 30	50	31	1		1	
Service year						
< 5	50	70	0.587 (0.362–0.951)*	0.030	0.760 (0.384–1.504)	0.431
≥ 5	84	69	1		1	
Working department						
Inpatient	107	104	1.334 (0.754–2.358)	0.332		
Outpatient	27	35	1			
Infection prevention practice						
Good	112	56	7.545 (4.271–13.329)*	0.000	7.313 (4.030–13.272)**	0.000
Poor	22	83			1	
Presence of instructive poster or guideline targeted on instrument processing in your department						
Yes	106	73	3.423 (2.008–5.833)*	0.000	2.675 (1.376–5.200)**	0.004
No	28	66	1		1	

*COR* Crude odd ratio, *AOR* adjusted odd ratio

* P-value < 0.05 crude, ** P-value < 0.05 adjusted

### Discussion

Failure to comply with the acceptable decontamination practice has a serious consequence [3, 20]. In effect, strict adherence towards the recommended decontamination practices by all healthcare workers is critical. Thus, this study aimed to assess the decontamination practice and associated factors among nurses working in Bale zone hospitals. This study showed that only 49.1% of the study participates had acceptable decontamination practice. In multivariable logistic regression analysis, reported good infection prevention practice and working in departments having instructive posters or guideline target on instrument processing were significantly associated with acceptable decontamination practice.

The finding implies that half of the study participants have unacceptable decontamination practice, which has been attributed as a potential problem in the fight against pathogenic microorganisms. This finding also comparable with other previous studies that reported a high prevalence of unsafe medical equipment disinfection among healthcare workers [10, 16].

In the present study, 77.3% of nurses reported they soak contaminated items in 0.5% chlorine solution before they perform any cleaning activity. And, 77.2% soaked within the acceptable time range. This finding is in agreement with the national infection prevention guideline recommendation [8].

Despite a strong theoretical basis and national recommendation to use appropriate personal protective equipment while performing decontamination activities only one out of five nurse use google/eye shield and 29.1% use mask while performing decontamination. This finding almost in agreement with a study conducted in Addis Ababa health centers; revealed that the level of personal protective equipment utilization while performing instrument processing was 22.6% [10].

In this study, nurses reported having good infection prevention practice were almost seven times more likely to had acceptable decontamination practice (AOR = 7.313; 95% CI: 4.030, 13.271, P = 0.000) than there counterpart. This association may be explained by the fact that those with good infection prevention compliance were exposed to basic instrument processing principles and skills therefore; they may achieve more acceptable decontamination practice. Further, working in departments having instructive posters or guideline targeted on instrument processing was the other predictor of acceptable decontamination practice. Nurses who were working in departments having instructive posters or guideline target was almost three times more likely to have acceptable decontamination practice than their counterparts (AOR = 2.675; 95% CI: 1.376, 5.200, p-value = 0.004); implying that availability of such guidelines has a positive effect on decontamination practice. In support of this, studies reported the positive correlation between availability infection prevention guidelines and healthcare workers good infection prevention compliance [13, 15, 21].

### Conclusions

This study indicated that only half of participates had acceptable decontamination practice, which is a great concern and might make hospitalized patients prone to different pathogenic microorganisms. Reported good infection prevention practice and working in the department having instructive posters or guideline target on instrument processing were factors positively associated with nurses acceptable decontamination practice. Therefore, providing educational training along with ensuring the availability of posters and/or guidelines targeted on medical equipment decontamination in each department may improve decontamination practice.

## Limitations

This study has a number of limitations; first self-reported results prone to social desirability bias, since healthcare workers might not give genuine responses to the self-administered questionnaire and commonly overstate their actual decontamination practice. Second, the study did not include observational data to validate the nurse’s decontamination practice. Finally, the study did not evaluate the thoroughness of cleaning, disinfection and sterilization practice.

## Additional file

**Additional file 1: Table S1.** Awareness of instrument decontamination of study participants in Bale zone hospitals in 2018 (n = 272).

## Data Availability

The datasets used during the current study are available from the corresponding author on reasonable request.

## Abbreviations

AOR: adjusted odds ratio
CI: confidence interval
SPSS: Statistical Package for Social Sciences
HAIs: healthcare-associated infections
