# Supplementary material for: Decontamination of patient equipment: nurses’ self-reported decontamination practice in hospitals of southeast Ethiopia
Source: BMC Res Notes. 2019 Jul 12;12:392. doi: 10.1186/s13104-019-4427-5 (PMC6624938; doi:10.1186/s13104-019-4427-5)
Supplement: Supplementary file 1 — Additional file 1: Table S1. Awareness of instrument decontamination of study participants in Bale zone hospitals in 2018 (n = 272). [file 13104_2019_4427_MOESM1_ESM.docx]

**Additional file 1: Table S1: Awareness of instrument decontamination of participants in Bale zone hospitals in 2018 (n = 272)**

| **Variables** | **Response** | **Frequency (n=273)** | **Percent (%)** |
| --- | --- | --- | --- |
| Is instrument processing is part of standard precaution component | Yes | 169 | 61.9 |
|  | No | 96 | 35.2 |
|  | I don’t know | 8 | 2.9 |
| Cleaning is the primary step in instrument processing activity | Yes | 100 | 36.6 |
|  | No | 171 | 62.6 |
|  | I don’t know | 2 | 0.7 |
| How do you prepare a 0.5% chlorine solution? | By mixing 9 part of water & 1 part chlorine solution | 240 | 87.9 |
|  | By mixing 10 part of water & 1 part chlorine solution | 25 | 9.2 |
|  | I don’t know | 8 | 2.9 |
| All microorganisms including spores can be destroyed by sterilization | Yes | 250 | 91.6 |
|  | No | 17 | 6.2 |
|  | I don’t know | 6 | 2.2 |
| Overall awareness of decontamination | Yes | 169 | 61.9 |
|  | No | 104 | 38.1 |
